# Gold Nanoparticles: A Powerful Tool to Visualize Proteins on Ordered Mesoporous Silica and for the Realization of Theranostic Nanobioconjugates

**DOI:** 10.3390/ijms19071991

**Published:** 2018-07-08

**Authors:** Marco Piludu, Luca Medda, Maura Monduzzi, Andrea Salis

**Affiliations:** 1 Department of Biomedical Science, University of Cagliari, Monserrato, CA 09042, Italy; 2 Department of Chemistry, CSGI, University of Florence, Sesto Fiorentino, FI 50019, Italy; medda.luc@gmail.com; 3 Department of Chemical and Geological Sciences, CSGI, University of Cagliari, Monserrato, CA 90042, Italy; monduzzi@unica.it

**Keywords:** ordered mesoporous materials, immunogold staining, gold nanoparticles, protein localization, nanomedicine, theranostics, nanobioconjugate

## Abstract

Ordered mesoporous silica (OMS) is a very interesting nanostructured material for the design and engineering of new target and controlled drug-delivery systems. Particularly relevant is the interaction between OMS and proteins. Large pores (6–9 nm) micrometric particles can be used for the realization of a drug depot system where therapeutic proteins are adsorbed either inside the mesopores or on the external surface. Small pores (1–2 nm) mesoporous silica nanoparticles (MSNs), can be injected in the blood stream. In the latter case, therapeutic proteins are mainly adsorbed on the MSNs’ external surface. Whenever a protein-OMS conjugate is prepared, a diagnostic method to locate the protein either on the internal or the external silica surface is of utmost importance. To visualize the fine localization of proteins adsorbed in mesoporous silica micro- and nanoparticles, we have employed specific transmission electron microscopy (TEM) analytical strategies based on the use of gold nanoparticles (GNPs) conjugates. GNPs are gaining in popularity, representing a fundamental tool to design future applications of MSNs in nanomedicine by realizing theranostic nanobioconjugates. It may be pointed out that we are at the very beginning of a new age of the nanomaterial science: the “mesoporous golden age”.

## 1. Introduction: Ordered Mesoporous Silica (OMS) Materials in Nanomedicine

### 1.1. The Beginning

**Figure 1:**
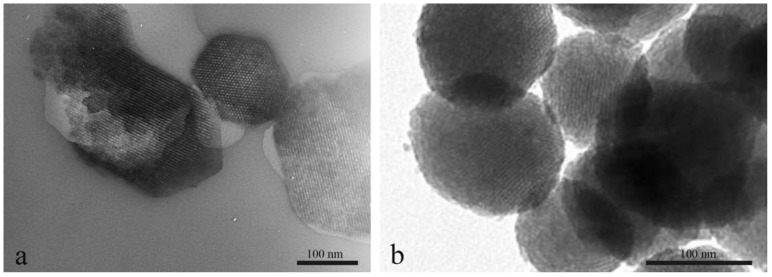
Transmission electron microscopy (TEM) images of Ordered mesoporous silica (OMS) nanoparticles. (**a**) Original SBA-15 materials and (**b**) MCM-41 based mesoporous silica nanoparticles (MSNs). Reprinted with permission from [10]. Copyright 2018, Elsevier.

As long ago as 1990, when they were discovered [1], ordered mesoporous silica (OMS) materials drew the attention of most material scientists and engineers. Due to their singular structural properties [2], OMSs have been intensely studied in recent years for the design of new biomaterials. They are mainly characterized by ordered channels and cavities with uniform pores (2–50 nm) [3] (Figure 1). The high surface area makes these materials suitable for new and innovative applications. OMSs have largely been investigated for catalysis [4] and biocatalysis purposes [5,6] and have shown potential features to be employed in nanomedicine [3,7,8,9]. As a result of the development of strategic synthesis procedures OMS structural features can be modified and fitted to specific aims, extending enormously their range of applications.

**Figure 2:**
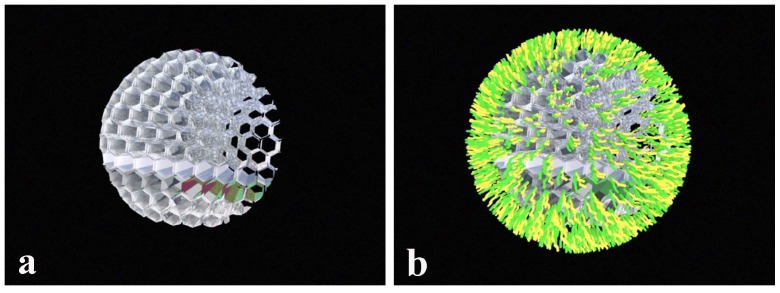
Schematic representation of the sequential steps of functionalization process of MSN surface with specific biomolecules. MSN particles before (**a**) and after (**b**) functionalization process.

Their capacity to house into the pore channels specific therapeutic molecules have paved the way for the developing of innovative applications of mesoporous silica as drug delivery systems [3,11,12,13,14,15]. OMS can be chemically modified to load and release specific therapeutic molecules according to controlled conditions [16,17]. Biomedical application of OMS is largely associated to their fate in biological media which, in turn, is related to their surface properties. Surface functionalization (Figure 2) plays a key role in determining biodegradation, cytotoxicity, and biodistribution through interactions which may be mediated by the macromolecules occurring in biological media [18,19,20].

**Figure 3:**
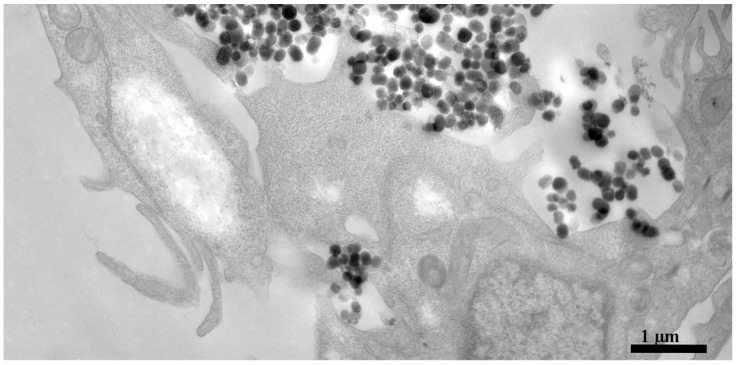
TEM micrograph of MSN interaction on Hela cell surfaces.

Important steps toward the development of targeted drug depot systems are the understanding of the mechanisms that regulate cell interaction of functionalized mesoporous silica nanoparticles (MSNs). Previous works have investigated by means of light microscopy the cellular uptake of MSNs in vitro. The strategy was to attach fluorescent dye molecules to the MSNs surface to visualize and locate the particles inside the cellular compartment [18,21,22,23,24,25]. Additional analyses through electron microscopy have highlighted, at ultrastructural level, the main morphological events that take place during cellular MSN internalization [18,25]. It was shown that cellular uptake of MSNs is strictly linked to the surface charge [25,26]. In this context, proteins have been indicated to be the right candidates to tailor the OMS behavior in a given biological system. For instance, surface protein functionalization of MSNs improves their biocompatibility when injected in biological media [27,28]. Moreover, the interaction between MSN and cellular surface can easily be modulated and controlled through specific protein functionalization [29] (Figure 3). Serum proteins, antibodies represent some of the wide variety of proteins that can be used to tailor chemical properties of MSNs in order to increase their biocompatibility or to set controlled release toward targeted tissues [30,31]. Particularly the adsorption of specific peptides and proteins characterized by important biological functions, represents key factors in the design and engineering of new nanosized drug-delivery systems. Moreover, it is well known that antibodies are biomacromolecules produced by immune system that are able to recognize a large variety of pathogens such as virus and bacteria [32] or to interact with cancer cells [33,34] through specific binding. In recent years, several therapeutic applications of the antibodies have been developed for the cure of autoimmune disorders or for the treatment of cancer disease [32].

### 1.2. The Golden Age

Several physico-chemical techniques, such as N_2_-adsorption isotherms and thermogravmetric analysis (TGA), provide important information on protein loading on OMS, but cannot directly visualize proteins adsorbed on the OMS’ surfaces. Different analytical methods are needed to deeply investigate the interaction of proteins with OMS and to elucidate the cellular interaction mechanisms of protein-MSN conjugates.

The strategic use of gold nanoparticles (GNPs) with dimension smaller than 100 nm has opened the way to the development of new and intriguing analytical procedures. The unique properties of GNPs make them suitable for biodiagnostic and bioanalitycal assays so that in the last years have received great attention as biosensors [35,36,37]. As a matter of fact, GNPs can be identified through different methodologies using their light absorption and scattering properties [38]. Because of their electron density properties GNPs are extremely suitable for labelling and can easily be revealed by electron microscopy techniques. Moreover, due to their peculiar surface chemistry they can be bound to a large variety of molecules, proteins included. All these features, added together, provide a formidable investigative tool to permit qualitative and quantitative analyses of protein location, and distribution at the ultrastructural level. Additionally, due to their optical features, they have been suggested for specific purposes that go beyond the imaging methodologies. Indeed, GNPs are good candidates for the development of theranostic nanobioconjugates to be used in medicine therapy [39]. It is well known that gold nanoparticles can be heated when exposed to light source. Photothermal therapy based on the use of the GNPs has already been tested in specifically targeted GNPs that can be used to destroy selectively malignant cancer cells, following laser exposure [40,41].

**Scheme 1:**
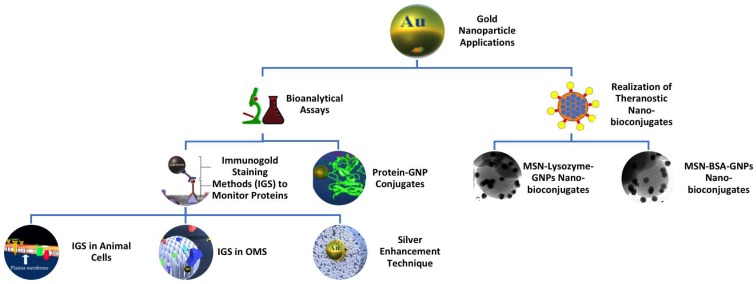
Diagram illustrating the main gold nanoparticle applications.

In this review, we aim to describe the principal innovative transmission electron microscopy (TEM) analytical strategies, based on the use of nanogold conjugates, to monitor proteins adsorbed on OMS. This topic is related to the exploitation of GNPs’ potentiality in different nanomedicine applications, spanning from controlled drug delivery depots to diagnostic and theranostic purposes (Scheme 1). This is just the tip of the iceberg.

## 2. “Seeing Is Believing”: Immunogold Labelling Methods to Monitor Proteins

### 2.1. Protein Localization in Animal Cells and Tissues: The Case of Histatin

Since their development immunogold staining (IGS) techniques became the elective procedures for macromolecules’ detection in animal cells and tissues. IGS techniques represent a fundamental tool in ultrastructural biological assays aimed to describe protein fine localization in animal tissues. IGS procedures were first developed by Faulk and Taylor in 1971 [42] to meet suitable protein analytical requirements during ultrastructural morphological investigations in animal tissues. They were able, for the first time, to improve the standard routinely TEM studies adding details at the molecular level about protein location and distribution. Before that, TEM methodologies were generally based on pure morphological analysis of biological samples. Shape, size, and number of subcellular organelles were the main data accessible following TEM examination. Since then, IGS techniques have extensively been used to localize proteins in animal tissues and revolutionized the way to investigate at ultrastructure level where morphological details were unequivocally combined to a significant amount of chemical data. The advantage of this methodology is the aptitude of gold-conjugated antibodies to recognize and bind specifically proteins, thus providing evidences about protein content and distribution. Colloidal gold, as electron dense marker, represents the best candidate probe to visualize and locate, by means of TEM, proteins exploiting the highly specific binding of antibody-GNP conjugates. GNPs provide an unequivocal signal that can easily be distinguished in both biological and inorganic samples.

**Figure 4:**
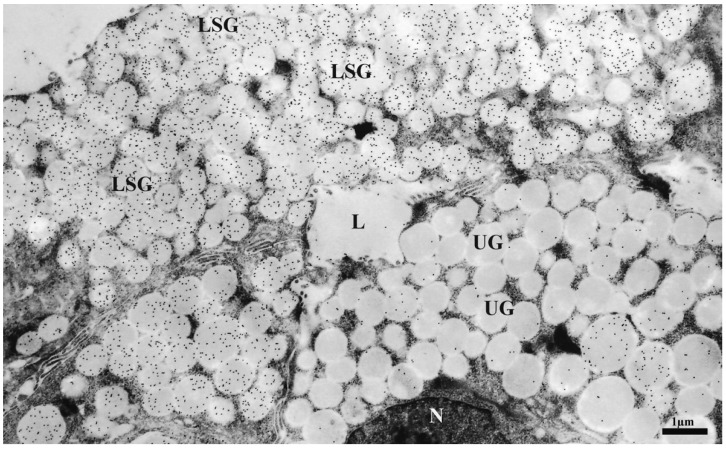
Electron micrograph of immunogold staining of ultrathin sections of human deep posterior lingual gland showing histatin localization. Gold nanoparticles are mainly visualized in the labelled secretory granules (LSG). Note that adjacent cells contain poorly or unlabelled granules (UG). L = lumen. N = nucleus. Reprinted with permission from [43]. Copyright 2006, Elsevier.

IGS was used by Piludu and coworkers to locate the presence and distribution of specific proteins in several animal tissues [43,44,45,46]. The purpose of these investigations was to provide additional data concerning the secretion process of different antimicrobial peptides (AMP) in human salivary glands. To date, the AMP have been well characterized during the last 20 years, however only a few data were reported about their localization and secretion in human tissues. The IGS procedures permitted, for the first time, to localize and to study the distribution of several AMP in human tissues and to definitely establish the involvement of the different human salivary glands in their production and secretion. In particular, the electron microscopic immunogold labelling extended enormously the previous biochemical investigations highlighting the fine visualization of AMP at the subcellular level (Figure 4).

### 2.2. Proteins Adsorbed on Ordered Mesoporous Silica Materials: The Case of Lysozyme

Although immunogold procedures were initially developed for biological assays, IGS was then employed for the functionalization of OMS materials to develop smart nanodevices for biomedicine applications. As previously stated, OMS materials have peculiar structural features that make them suitable hosts to immobilize into their pores a large variety of macromolecules with important biological functions [16]. Among the OMS materials, SBA-15 has largely been studied as a potential carrier for different purposes. Previous investigations studied the interaction and adsorption mechanisms of several therapeutic molecules onto SBA-15 [3,47,48]. These studies pointed out the potentiality for obtaining smart delivery systems. Interestingly, specific chemical surface functionalization of SBA-15 was shown to enable peculiar biocompatibility conditions [10,18,49].

**Figure 5:**
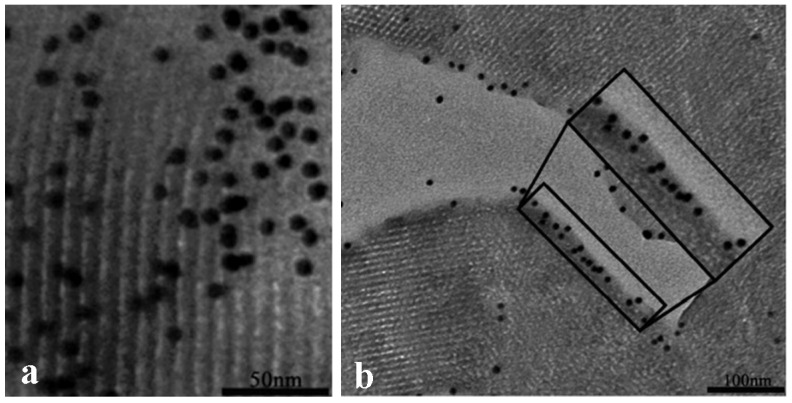
TEM immunogold labelling of ultrathin sections (~60–80 nm thick) of SBA-15 with anti-lysozyme antibody. Gold labelling is detected into cylindrical channels (**a**) and on the surface of SBA-15 (**b**). Reprinted with permission from [50]. Copyright 2011, Royal Society of Chemistry.

In order to gain direct visualization of proteins adsorbed onto SBA-15 materials, we used for the first time the IGS techniques in our investigations [50]. We studied the location and distribution of the human lysozyme adsorbed onto mesoporous silica SBA-15 particles using a post embedding immunogold staining procedure. The advantage of this method is due to the high resolution of the gold labelling, and its unambiguous electron dense mark that can simply be evidenced by transmission electron microscopy. Human lysozyme loaded and unloaded SBA-15 microparticles (around 300 nm) were embedded in LR gold resin blocks. After resin polymerization, the embedded samples were cut into ultrathin sections (90 nm thick), and then processed for immunogold staining through two consecutive incubation steps of the sections with specific antibodies. The first step of this procedure was characterized by the interaction of a primary antibody, specific to human lysozyme, whereas in the following step a gold-conjugated secondary antibody, specific to primary antibody, was added. TEM analysis highlighted the presence of specific lysozyme reactivity that was visualized as definite black spots into cylindrical channels or located on the surface of SBA-15 particles (Figure 5).

**Figure 6:**
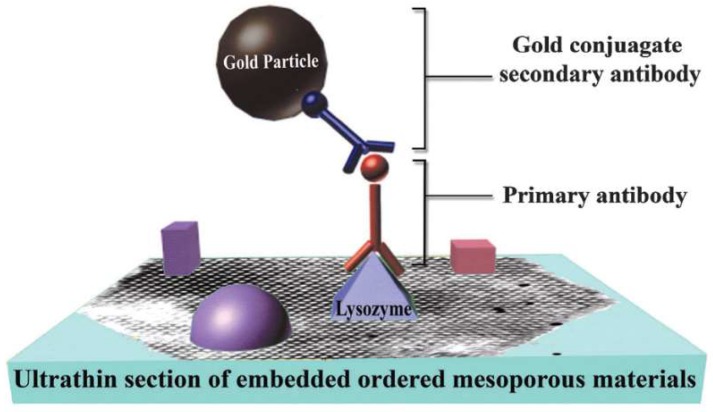
Schematic representation of post embedding immunogold staining (IGS) procedure for protein visualization by conventional TEM. Note that purple and red objects represent additional proteins adsorbed onto OMS. Reprinted with permission from [50]. Copyright 2011, Royal Society of Chemistry.

The selection of an appropriate antibody in immunolocalization assays is extremely crucial to gain reliable results, in particular, considering that choices have to be made on the basis of availability, quality of antibodies and specificity towards a given protein. The achievement of significant results in immunolocalization investigations deals with the aptitude of the primary antibody to bind specifically proteins through an important biochemical interaction that leads to the formation of the antibody-antigen complex [51]. The present method, being characterized by the sequential binding of primary, and secondary antibodies, is generally known as indirect method. Indeed, the gold marker is associated to the primary antibody-antigen complex through linking the secondary antibodies (Figure 6).

In order to meet peculiar needs of immunochemical assays different IGS procedures can be used. Remarkably, the indirect method in the recent years has gained in popularity compared to the direct IGS procedure that is based on the GNPs directly bound to the primary antibodies. The reason for that may be found in the higher sensitivity of the indirect method as a result of the capacity of secondary antibodies to interact with multiple epitopes located on primary antibodies [51,52]. Because of the sequential steps of incubations with primary and secondary antibodies, it should be noticed that indirect methods may imply the concurrence of cross-reactions with unrelated molecules [51]. It follows that suitable and appropriate control samples are of utmost importance. In our investigation we performed specific controls in order to verify the specificity of lysozyme labelling in loaded SBA-15. Indeed, the failure of antibody performance may occur at any stage of the procedure. During our investigations unloaded samples of SBA-15 were incubated with both primary and secondary antibodies to ascertain antibody specificity. Moreover, additional controls were performed by incubating human lysozyme loaded samples with non-immune serum or with the secondary antibody only and omitting the primary antibody (negative control). No gold labelling was detected in any of these cases [50].

Peculiar modifications of the standard IGS protocol regards the use of multiple sequential staining procedure of two or more proteins in the same sample, using different size gold conjugated antibodies. One of the main advantages of the double immunostaining procedure concerns the opportunity to study simultaneously several proteins adsorbed in the same material. Choosing different gold nanosized conjugated secondary antibodies specific for the primary antibodies allows to monitor the different proteins separately. This technique, successfully applied in several histochemical studies, involves the use of different size nanogold conjugated antibodies that can easily be distinguished during TEM analysis, thus allowing to visualize spatial relationships of two or more proteins in the same section.

### 2.3. Silver Enhancement Technique and Ultra-Small Gold Nanoparticles: The Case of an Antibody Fragment

Small GNPs (gold size ≈ 1–3 nm) were firstly introduced in order to improve the penetration of gold conjugated antibodies into animal cells and tissues and to enhance labelling efficacy [53,54]. In pre-embedding immunocytochemical assays gold conjugated antibody efficiency deals with the size of gold particles used. It was shown that the antibodies employed in routinely IGS procedures with gold size particles around 10–30 nm show reduced interaction efficiency. Instead, smaller nanoparticles were shown to be characterized by a higher degree of penetration, improved gold conjugated antibody interaction with the targeted antigens and higher resolution of labelling [54,55]. Because of their properties ultra-small GNPs may be used in high resolution labelling for protein detection in nanostructured materials and in the development of targeted drug delivery systems. However, the ultra-small GNPs cannot be visualized by conventional TEM but need to be amplified and converted in larger size particles through silver enhancement procedure [56].

**Figure 7:**
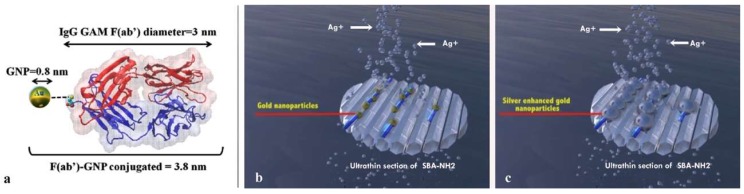
(**a**) Representation of the antibody fragment-gold nanoparticle [F(ab’)GAMIgG-GPNs] conjugate; and (**b**,**c**) Graphical representation of the main sequential steps of silver enhancement process on ultrathin section of F(ab’)GAMIgG-GPNs loaded SBA15. (**a**) is adapted with permission from [57]. Copyright 2015, American Chemical Society.

Similarly, we used the silver enhancement procedure to visualize the antibody fragments F(ab’)GAMIgG conjugated to ultra-small gold nanoparticles (GNPs diameter 0.8 nm) immobilized into the mesopores of amino functionalized SBA-15 particles [57]. The silver-enhanced GNPs resulted visible at conventional TEM, thus allowing for an unequivocal imaging of the location of the antibody fragment-GNPs conjugates inside the channels of SBA-15 particles (Figure 7).

**Figure 8:**
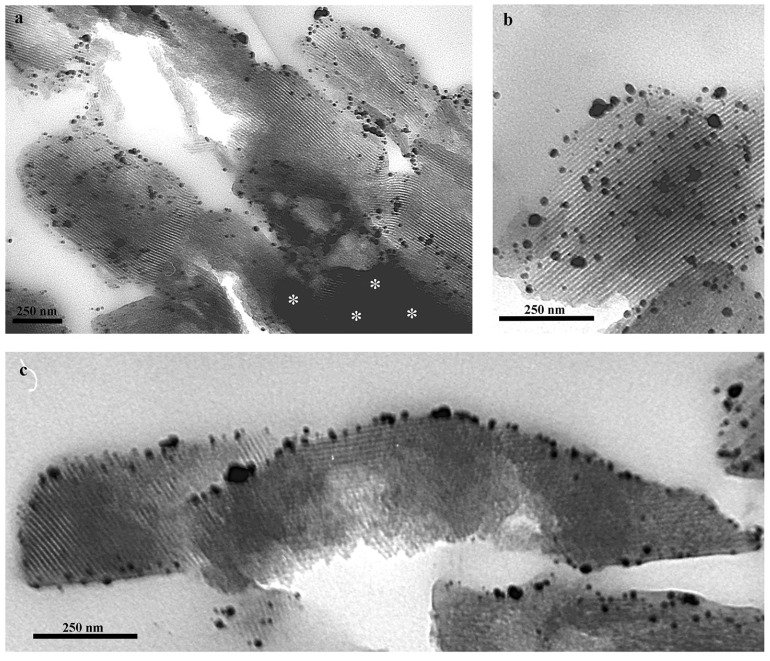
Transmission electron micrographs of ultrathin sections of SBA-15 particles loaded with F(ab’)GAMIgG-GNPs conjugates. Distribution of gold conjugated F(ab’)GAMIgG in SBA-15 particles was revealed by silver enhancement procedure. Black spots correspond to the “silver enhanced” GNPs (silver labelling). (**a**) Overview of silver localization in loaded SBA-15 samples. Note that SBA-15 particles show variable labelling. Dark area (asterisks) is due to the electron beam passing through a thicker portion of SBA-15 section; (**b**) Details showi ng at higher magnification the presence of silver labelling along the parallel channel of loaded SBA-15; and (**c**) Silver labelling is confined to the external portion of SBA-15 particles. Reprinted with permission from [57]. Copyright 2015, American Chemical Society.

The use of sub-nanometer gold increased enormously its diffusion and penetration efficiency due to the reduced overall size and decreased steric hindrance. This procedure was carried out by embedding both F(ab’)GAMIgG-GNPs loaded and unloaded SBA-15 micro-particles in resin, following standard preparation procedure [58] and finally cutting ultrathin sections (70–90 nm thick) of both embedded samples with ultramicrotome. The ultrathin sections were finally treated through the silver enhancement procedure. In detail, the silver enhancement technique permitted the amplification of the ultra-small GNPs that acted as nucleation sites for the deposition of Ag atoms obtained through the reduction of Ag^+^ ions in all loaded samples of SBA-15. The unloaded samples used as controls were devoid of labelling. The TEM observation revealed the presence of unequivocal silver labelling as 20–30 nm size black dots on the mesoporous inner structure. The black dots highlighted the presence and distribution of antibody fragments F(ab’)GAMIgG either inside the pores or adsorbed on the surface of SBA-15 microparticles (Figure 8).

On the basis of these results, it should be remarked the importance of this powerful procedure to study and gain site-specific data concerning the functionalization process of specific peptides and proteins in OMS materials, and thus in the engineering of new drug delivery systems. Moreover, the use of ultra-small gold particles represents the best choice for molecular labelling, since it does not seem to prevent the penetration of the proteins into the pore channels of SBA-15, considering that the silver labelling is highly specific and highly sensitive towards the gold particles [59].

## 3. Exploring the Formation Mechanism of the Protein Corona: The Case of BSA-GNP Conjugates

**Figure 9:**
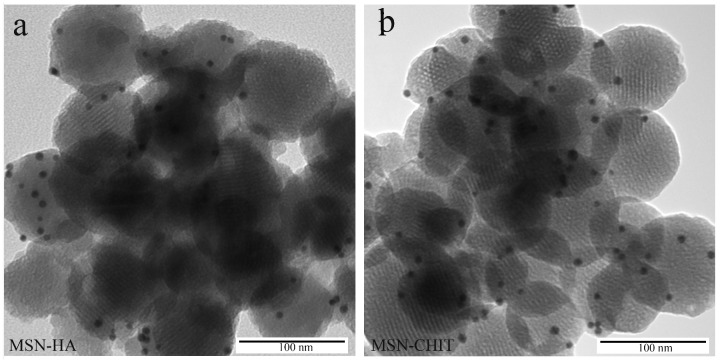
TEM micrographs of MSN-HA+BSA-GNPs (**a**) and MSN-CHIT+BSA-GNPs (**b**). Reprinted with permission from [10]. Copyright 2018, Elsevier.

Gold nanoparticles can be bound directly to a large variety of biomolecules by using definite chemical methodologies. For instance, hydrophobic interactions or charge pairing represent the main procedures employed to conjugate GNPs to specific molecules. During our investigations we used gold complexes to study the adsorption process of biomolecules on the surface of functionalized mesoporous silica nanoparticles (MSNs) dispersed in biological media. In detail, we investigated the interaction of gold conjugated bovine serum albumin (BSA) with hyaluronic acid (HA) and chitosan (CHIT) functionalized MSNs to provide new insights on the molecular mechanisms that lead to the formation of the surface layer of biomolecules around the MSN, known as the “protein corona” [10]. Protein corona induces significant changes in the physico-chemical properties of MSNs surface. This in turn may affect MSNs cellular interaction and uptake. Protein corona formation is influenced by both chemical MSNs features and composition of the biological fluids. It has been reported that functionalized MSNs, when injected in biological media, are characterized by the interaction of their surface with the dispersed biomolecules. This process is supposed to be initiated by specific physico-chemical interactions that rely mainly on the peculiar MSNs features such as surface charge [60,61]. To this purpose, the behavior of BSA interaction with the differently functionalized MSNs was investigated by conventional TEM analysis. We used BSA conjugated with gold nanoparticles (BSA-GNPs). Evident gold labelling was observed on the surface of both MSN-HA and MSN-CHIT samples, that were characterized by similar BSA-GNPs patterns. Occasionally, higher gold labelling intensity was found in MSN-CHIT than in MSN-HA, pointing out that BSA interaction depends on the molecular features of the biopolymers adsorbed on MSN surface (Figure 9).

Here, it should be remarked that, at a pH around 7, BSA carries a negative charge (being pI ≈ 4.7), whereas MSN-CHIT and MSN-HA surfaces are positively and negatively charged, respectively. Indeed, protein adsorption is mainly addressed by electrostatic interactions according to the order MSN-CHIT > MSN-HA; however, the relevant BSA adsorption on the negatively charged MSN-HA implies important contributions of van der Waals attractive interactions, as demonstrated by zeta potential data (see Table 2 in [10]). A similar trend was also observed after the gold labelling procedure. The GNP conjugation provided a powerful methodology for the direct visualization of interaction process of BSA with functionalized MSN surfaces, correlating surface characterization of MSNs with BSA adsorption. In other words, the formation of a protein corona around functionalized MSN particles, independently of the electrostatic forces, was unequivocally proven [10].

## 4. Toward the Realization of Theranostic Nanobioconjugates

**Figure 10:**
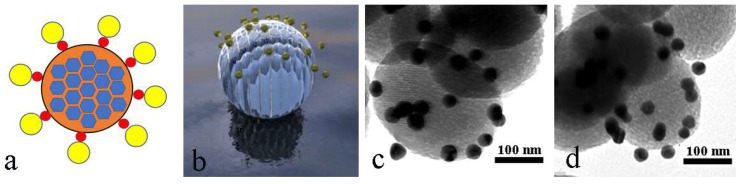
Assembly of theranostic nanobioconjugates. Schematic representation of the MSN-protein-GNP nanobioconjugate: MSN (orange/blue), protein (red), GNP (yellow) (**a**), graphical 3D reconstruction of the MSN-protein-GNP nanobioconjugate (**b**), TEM micrographs of the MSN-lysozyme-GNP (**c**) and MSN-BSA-GNP (**d**) nanobioconjugates.

In the previous paragraphs we have described the use of GNPs as diagnostic devices for the localization of proteins within the internal and the external surface of OMS. Besides diagnostic imaging, GNPs may find different application in nanomedicine. GNPs are indeed characterized by a surface plasmon frequency in the visible range, which makes them suitable for therapeutic treatments (i.e., photothermal therapy [62] or, more interestingly, for “theranostics” that is the combination between therapy and diagnostics [63]. In a very recent work [64], we assembled multicomponent nano-bioconjugates based on mesoporous silica nanoparticles (MSNs), proteins (BSA or lysozyme), and gold nanoparticles (Figure 10). The purpose of the realization of such nano-bioconjugates was for a possible application in nanomedicine as theranostic devices. Indeed, MSNs can act as drug carriers, proteins stabilize MSNs within the bloodstream, or may have therapeutic or targeting functions. Finally, GNPs could either be used as contrast agents for imaging or for photothermal therapy. The synthesized MSNs, functionalized with amino-terminated groups (MSN-NH_2_), were conjugated to BSA or lysozyme on the external surface of MSN-NH_2_ to obtain MSN-BSA and MSN-Lysozyme bioconjugates, respectively. The MSN-protein samples were dispersed in a GNP solution to obtain MSN-protein-GNPs nano-bioconjugates. TEM analysis showed the occurrence of GNPs on the MSN-protein surface. Interestingly, we demonstrated, through fluorescence and Raman measurements, that GNP-protein specific interaction occurs through the involvement of tryptophan amino acid residues. Hence, the MSN-protein-GNPs nano-bioconjugates formed in the presence of lysozyme display more conjugated GNPs than those formed in the presence of BSA: This was related to the higher number of tryptophan residues in lysozyme compared to BSA.

## 5. Conclusions

The use of GNPs represents one of the most encouraging strategies to monitor proteins in many research fields. They have been suggested as biosensors because of their optical properties and are excellent labels that can be detected through several procedures. GNPs can be conjugated with a large variety of biomolecules. Because of their comparable size with proteins, peptides, oligonucleotides, and other biomolecules, GNPs can be used in many biomedical application fields. They can be used as drug carriers to deliver specific therapeutic molecules as a result of their ability to link and load different molecules such as peptides, proteins, or oligonucleotides [65,66,67]. The GNPs, when conjugated to specific targeting proteins, can also be used as intrinsic drug agents. Their capacity to penetrate cellular compartments and induce cellular damage by cellular oxidative stress, or by photothermal therapy has been used to ablate specifically cancer cells and tissues. However, even though their use as contrast agents remains the main application in imaging and diagnostic medicine, it should be remarked that GNPs based approaches are becoming a fundamental tool in the development of new theranostic nanobioconjugates. The main aim of this review was to bring the attention of the readers to the extraordinary high resolving power of the gold labelling techniques, building a bridge with the other investigative physical and chemical disciplines. This may provide new eyes to the way how we look at the nanostructured world of the ordered mesoporous silica materials.

## Abbreviations

OMS	Ordered mesoporous silica
MSN	Mesoporous silica nanoparticle
GNP	Gold nanoparticles
IGS	Immunogold staining
AMP	Antimicrobial peptides
BSA	Bovine serum albumin
HA	Hyaluronic acid
CHIT	Chitosan
